# Clinicoradiological Predictors of Severity of Traumatic Intra-Abdominal Injury in Pediatric Patients: A Retrospective Study

**DOI:** 10.7759/cureus.17936

**Published:** 2021-09-13

**Authors:** Garima Sharma, Navojit Chatterjee, Ashish Kaushik, Sudhir Saxena

**Affiliations:** 1 Department of Radiology, All India Institute of Medical Sciences, Rishikesh, IND

**Keywords:** trauma pediatric, abdominal trauma, computed tomography (ct ), fast, pediatric trauma protocols

## Abstract

Background

Adequate assessment of traumatic injury in patients of all age groups is essential for timely intervention and prevention of mortality and morbidity. This study aimed to assess the value of certain clinical as well as radiological factors as predictors of severity of the intra-abdominal injury as detected on computed tomography (CT) and to review the guidelines, protocols, and practices followed in imaging of abdominal trauma in patients of pediatric age group.

Methods

This retrospective observational study included 263 pediatric patients (18 years of age or younger) who presented to the emergency department (ED) with a history of trauma to the abdomen. The study was conducted over a period of 12 months. Correlation of five variables, i.e., age of the child, focused abdominal sonography in trauma (FAST) status, mechanism of injury, presenting complaints and clinical features (hypotension, tachycardia, etc), fractures identified on trauma X-ray series, was done with CT findings (severity of injury). All five variables were statistically analyzed and p-values were derived for age, mechanism of injury, presenting complaints, clinical features, and trauma x-ray series, while parameters like sensitivity and specificity were determined for FAST status

Results

All variables well correlated with the severity of injury with p-values <0.05. On multivariate analysis, FAST status had the highest (47.94) odds ratio among the five variables for predicting severe intra-abdominal injury while vital signs had the lowest (0.076). Further, age group of 0-4 years was found most prone to higher grades of injury with odds ratio of 7.83. Motor vehicle crash had odds ratio of 26.6 for severe injury, the highest among mechanisms of injury. While for FAST status, sensitivity was found to be 89.4%, specificity 85%, and negative predictive value 90%, trauma series radiographs had a sensitivity of 42.27%, specificity of 77.85% and negative predictive value of 60.55%.

Conclusion

Clinical parameters and traditional imaging techniques can predict the severity of injury on CT and guide further imaging and intervention.

## Introduction

Trauma is one of the leading causes of morbidity and mortality among children. Often these accidents are unobserved and the clinical evaluation of children with an abdominal injury presents a challenging task. Although there are several pre-hospital triage tools and clinical prediction rules [1], the situation is often tumultuous to conclude a serious intra-abdominal injury based on these. Most ED physicians consider whole-body CT as the safest and fastest method for assessment of multisystem injury including abdominal injuries. While CT is an excellent tool for complete assessment and documentation of the extent of all major injuries, it comes with its demerits, mainly radiation exposure.

The pattern of injury in children differs from adults in many ways [2], hence the approach to imaging and diagnosis should be modified accordingly. Current imaging and diagnostic guidelines by various trauma societies and injury classification systems mainly apply to adults patients with minimal modifications for the pediatric age group [3-5]. This study investigates the utilization of CT study for assessment of abdominal injuries in pediatric age group patients, imaging guidelines by different trauma societies, and assessment of certain clinical parameters and non-ionizing imaging methods as predictive factors for severity of the injury.

## Materials and methods

Study design

We conducted a retrospective observational study at a tertiary level trauma center for a period of 12 months (June 2019-June 2020)

Selection of participants

We included patients younger than 18 years of age with a history of blunt trauma to the torso and underwent, trauma series X-rays which include the lateral cervical spine, a supine anteroposterior (AP) chest X-ray (CXR), extremities, and an AP pelvis X-ray, FAST and contrast-enhanced CT scan for assessment of the injury. Patients were managed in the ED according to the advance trauma life support (ATLS) protocol. 

Five study variables were collected retrospectively and correlated with the severity of the injury as detected by a CT scan. Patients with unstable vital signs who responded to fluid resuscitation (transient responders according to ATLS protocol) were included in the study while non-responders and those requiring immediate operative management were excluded from the study. Also, children with incomplete study variables, e.g. fewer X-rays, unknown FAST status, were excluded from the study.

Data collection and processing

Data were collected in a retrospective manner. The history and physical examination findings included the age, mechanism of injury, clinical signs, and parameters (systolic blood pressure and pulse rate adjusted to age). In addition, we included the finding of FAST examination and radiographs (chest, extremities, etc). Hence, a total of five variables were evaluated.

Outcome measures

The primary outcomes were the presence of intra-abdominal injury. The intra-abdominal injury was defined as an injury to any of the abdominal structures and graded according to the American Association for the Surgery of Trauma (AAST) injury scoring scales for any organ injury. These were categorized into three groups as follows: Group 1 = grade IV or higher in at least one organ, Group 2 = grade III or lower injury in at least one organ, Group 3 = negative/normal CT study.

Data analysis

Clinical detailed and physical examination details were assessed by a senior ED physician. All radiological investigations were assessed by three radiologists. For age, logistic regression was used to find the correlation with the severity of the injury. The other four variables were analyzed by Chi-square test and p-values were derived for them. Parameters like sensitivity and specificity were determined for FAST status and radiographs. Odds ratio were calculated for the variables with a 95% confidence interval.

## Results

After applying the exclusion criteria, we analyzed 263 patients presenting with a traumatic abdominal injury. CT showed the presence of grade IV or higher injuries in 15% (n=39) patients, grade III or lower injuries in 32% (n=84) patients. Negative CT was seen in 53% (n=140) patients.

The patients’ ages were subdivided in five-year units into four strata (0-4, 5-9, 10-14, and ≥14 years) to examine differences of injury severity relative to age. The mean age of presentation was 11 years. The regression coefficient was -0.657 with p = 0.009 indicating lower age groups had greater severity of the injury. 

Clinical assessment done in ED included presenting features like abdominal wall ecchymosis, abdominal tenderness, persistent per rectal or nasogastric bleeding. Clinical parameters like hypotension (systolic blood pressure) and/or tachycardia were taken into account. Two patients presented with unstable vitals and responded to resuscitation at the ED (transient responders), had grade IV and higher injuries on CT scan (Table 1).

**Table 1:** The presenting features and vital stability in the subjects. ^#^Responding to fluid resuscitation (transient responders). *Abdominal wall ecchymosis, abdominal tenderness, persistent per rectal or nasogastric bleeding.

Clinical variables	Severity of injury			
		Normal, N(%)	Grade III or below, N(%)	Grade IV or above, N(%)
Vitals (blood pressure and/or pulse rage adjusted for age)	Stable	140(100)	83(98.8)	35(89.7)
	Unstable^#^	0(0)	1(0.2)	4(10.7)
Total		140	84	39
Presenting features*	Present (1 or more)	31(22.1)	28(33.3)	24(61.5)
	Absent	109(77.8)	56(66.7)	15(38.5)
Total		140	84	39

All the children underwent FAST examination. FAST status was categorized as (i) normal or negative examination, (ii) fluid demonstrated in one pocket, i.e., (pleural, pericardial, hepatorenal, perisplenic, or pelvic regions) and (iii) fluid demonstrated in more than one pocket. 85% (n=119) patients who had negative CT study also had FAST negative status, while none of the patients with grade IV or higher injury had negative FAST study (Table 2).

**Table 2:** FAST status of the subject. FAST: focused abdominal sonography in trauma.

FAST status	Severity of injury		
	Negative, N(%)	Grade III or below, N(%)	Grade IV or above, N(%)
Negative	119(85)	13(15.4)	0(0)
Fluid in one pocket	21(15)	28(33.3)	2(5.2)
Fluid in more than one pocket	0(0)	43(51.7)	37(94.8)
Total	140	84	39

Considering the mechanism of injury, maximum number of patients present with motor vehicle crashes 40.7% (n=152) (Table 3). Other mechanisms include fall from height/stairs, object striking abdomen, and unknown causes.

**Table 3:** Different mechanisms of injury in presenting subjects.

Mechanism of injury	Severity of injury		
	Normal, N(%)	Grade III or below, N(%)	Grade IV or above, N(%)
Motor vehicle crash	57(40.7)	70(83.3)	25(64.1)
Fall from height	47(33.5)	7(8.3)	10(25.6)
Object striking abdomen	20(14.2)	6(7.1)	4(10.3)
Unknown	16(11.6)	1(1.3)	0(0)
Total	140	84	39

All included children underwent an initial radiographic study (trauma series) for assessment of bony injuries. Less percentage of patients who had head, facial, or extremity fractures had grade IV injuries (Table 4).

**Table 4:** Findings of trauma X-ray series.

Other radiological study	Severity of injury		
	Negative, N(%)	Grade III or below, N(%)	Grade IV or above, N(%)
Fractures involving torso:			
Fractures of pelvis	10(7.1)	5(5.9)	2(5.1)
Fracture of ribs	28(20)	2(2.4)	0(0)
Fracture not involving the torso (extremities, head or face)	69(49.3)	77(91.7)	29(74.3)
No fracture identified	33(23.6)	0(0)	8(20.6)
	140	84	39

All variables were well correlated with the severity of injury with p-values <0.05. To determine the predictive value of variables, a multivariate analysis was done for each (Table 5). For age, >14 years age group was set as the reference, the odds ratios for incurring a severe injury was highest in 0-4 years group (7.83). A decreasing trend in the odds ratio was seen with increasing age, indicating younger children are more prone to severe injury as compared to older ones. For mechanism of injury, after setting unknown causes as reference, it was observed that motor vehicle crash had the highest odds ratio for severe intra-abdominal injury (26.66), followed by object striking the abdomen (8.000). While vital signs had lowest odds ratio among the five variables (0.0767), FAST status had the highest odds ratio (47.94) for predicting severe injury. Odds ratio of 4.43 was observed for trauma series radiographs.

**Table 5:** Multivariate analyses for five variables. 95% CI: 95% confidence interval.

Variable		Odds ratio	95% CI	p-value
Age (in years)	0-4	7.833	3.5272-17.3967	<0.0001
	5-9	3.3791	1.6715-6.8311	0.0007
	10-14	1.1463	0.5276-2.4909	0.0053
	>14	Reference	Reference	
Mechanism of injury	Motor vehicle crash	26.6667	3.4438-206.4895	0.0017
	Fall from height	5.7872	0.7122-47.0282	0.0005
	Object striking abdomen	8.0000	0.9242-69.2462	0.0390
	Unknown	Reference	Reference	
Vital signs		0.07667	0.00419-1.4011	0.0083
Presenting features		2.5752	1.5069-4.4008	0.0005
FAST status		47.9487	22.90-100.36	<0.0001
Trauma x-ray series examination		4.4332	1.96604-10.0.26	0.0003

For FAST status, sensitivity was found to be 89.4%, specificity 85%, and negative predictive value 90%. Trauma series radiographs showed sensitivity of 42.2% and specificity of 77.8%, negative predictive value 60.5%. 

## Discussion

A child presenting to ED with a history of trauma is a situation of greater concern for the attending physicians as compared to adult patients. The victim is irritable and parents are anxious, often disproportionately relative to the severity of the injury, which makes overall assessment much more difficult.

There are significant anatomical, physiological and psychological differences between adult and pediatric patients. Greater distribution of traumatic forces results in multiple injuries, less subcutaneous fat and musculature predisposing to internal organ injuries, greater head to body ratio leading to serious head injuries, and growth plate injuries are few to mention [2]. Also, they have a greater capacity to maintain blood pressure despite the acute blood loss. Even subtle changes in these parameters must alert the attending physician about such injuries.

Although the role of pre-hospital assessment cannot be completely overlooked, these tools remain unreliable in the prediction of the severity of the injury. In a study by Cheung R [1], eight pre-hospital pediatric triage tools were identified and assessed for identifying serious injury in injured children. Many of them demonstrated both unacceptable over and under-triage rates.

Guidelines for imaging severely injured patients of any age stress the need for imaging protocols with high sensitivity and specificity. While Pediatric Emergency Care Applied Research Network (PECARN) and National Institute for Clinical Excellence (NICE) guidelines have been developed for the imaging of head and neck trauma while limiting radiation exposure, no comprehensive guidelines are available till date for imaging of the chest, abdominal and pelvic injuries. The pediatric trauma protocol given by the Royal College of Radiologists (RCR) in 2010 [3], states that a pediatric patient should be referred to a pediatric radiologist for appropriate imaging protocols. What is the appropriateness, however, is not indicated. Also, there is a lack of pediatric radiologists at most centers, which is another great limitation of this protocol. Few clinical variables like the "seat belt sign"[6,7], nasogastric, or per rectal bleed which may need CT evaluation are mentioned according to RCR protocol. 

The pediatric trauma society indicates the use of CT in cases of falling hemoglobin on serial examination and symptomatic patients, in suspected cases of liver or splenic injuries [5].

Several studies have been conducted in past investigating the performance of various clinical parameters and laboratory as well as non-ionizing radiological investigation in predicting the severity of injury in patients. In series of studies with internal as well as external validation conducted by Holmes JF et al [8-10], a combination of clinical findings was found useful to determine which patients required further evaluation. Also, the role of laboratory tests in the evaluation of pediatric trauma patients has been studied, however, is considered controversial. The study stated that the application of the prediction rule accurately identified children at very low and high risk for intra-abdominal injury and would have significantly reduced abdominal CT use by 33% [10]. Ashrafi et al [11] showed in their study that a combination of three ultrasounds, urine analysis and liver function tests can predict intra-abdominal injury and reduce the cost of diagnostic workup. In another study, Cotton et al [12] assessed the utility of 23 clinical variables potentially associated with intra-abdominal injury in pediatric age group.

Many studies consider whole-body CT as the most appropriate imaging modality, supporting its continued use in the initial assessment of trauma patients [1] and several others validated the same [13,14]. Several factors are often undermined, the first and the most important being radiation exposure. The relationship between ionizing radiation exposure during imaging and the risk of development of malignancies has been well documented by various studies [15-19]. According to the study conducted by Pearce MS et al., the use of CT scans in children deliver cumulative doses of about 50 mGy might triple the risk of leukemia, and doses of about 60 mGy might the risk of brain cancer to three times [17]. The second factor is cost. Routine use of CT scan also raises the overall cost of health care. Other factors are availability, need for contrast injection, sedation, and follow-up scans.

Another consideration about CT imaging is the multiphase examination for identification of any active vascular bleed. Although, dual-phase contrast-enhanced study which ensures maximal synchronous enhancement of both arterial and venous systems is now widely recommended, at centers like ours, the practice of acquiring separate arterial and venous phases is still practiced. Sometimes, a delayed phase is also recommended for the assessment of urinary tract injuries. These examinations double the radiation dose to the patient. Also, children have an enhanced vasoconstrictor response, therefore, imaging protocols with separate arterial and venous-enhanced examinations may falsely reassure the attending clinician [20]. Clinical observation to look for hemodynamic instability in such cases becomes the only cue to diagnose acute bleeding.

The primary goal of our study is to emphasize the role of clinical parameters and traditional radiological investigation which can assist both ED physician as well as the radiologist in better assessment of pediatric patients. We considered these variables because these are readily available to the attending physician at the patient's bedside, unlike laboratory investigations which take time to process and produce results.

While age and mechanism of injury are very well correlated with the severity of the injury, mechanism of injury alone cannot be the guiding indication for a CT scan [21]. With CT being an excellent tool to detect skeletal injuries, traditional trauma series radiographs have been largely replaced [22,23]. As also seen in our study, pelvic radiograph has poor sensitivity for predicting severe injuries [24]. Their roles could be made important in combination with clinical as well as sonological examination.

In our study, FAST has an excellent negative predictive value and high sensitivity. Studies have shown that FAST has a sensitivity of 70% and specificity of 100%, but when combined with physical examination, the sensitivity can rise to 100% [25,26]. Expanding the scope of this modality to detect visceral injuries and better training of performing radiologists/physicians are important steps in curbing the overuse of CT.

Presenting clinical features had the lowest correlation with the severity of the injury. This can be attributed to non-uniform triage methodologies and heavy reliance on imaging for injury assessment.

In summary, all the five variables together identified most patients with severe intra-abdominal injuries. Prospective application of this clinical assessment can obviate unnecessary CT examinations as well as serve as guides for further imaging assessment of the patient.

## Conclusions

Increased use of CT examinations is observed in the majority of trauma centers catering to pediatric patients. More than 50% of scans in our study were negative/normal, indicating a need for risk stratification based on various clinical and radiological factors. Age as a factor showed a negative correlation with the severity of the injury, while the rest of the variables showed a significant statistical correlation with the severity of the injury. While our study was able to show that clinical indicators of severe traumatic injury can predict radiologic findings such that more severely injured children are more likely to have injuries that can be detected on imaging, which is an obvious conclusion. However, the corollary of this conclusion holds greater importance, such that, patients with clinical indicators of less severe injury are less likely to have a radiologically severe grade of injury. Hence, imaging requirements should be evidence-based, to minimize the IR burden to this sensitive population. Majority of the traumatic injuries in pediatric patients are managed conservatively. The need to diagnose an otherwise low-grade injury should not take precedence over the appropriateness of imaging and the risk-benefit ratio of imaging. Thorough and careful clinical examination and observation are keys for the proper risk stratification of children presenting to the ED with a history of trauma, which is the responsibility of both the ED physician and the radiologist.
